# Supplementary material for: Nb-induced lattice changes to enhance corrosion resistance of Al0.5Ti3Zr0.5NbxMo0.2 high-entropy alloys
Source: Nat Commun. 2025 Mar 22;16:2828. doi: 10.1038/s41467-025-58211-9 (PMC11929843; doi:10.1038/s41467-025-58211-9)
Supplement: Supplementary file 1 — Supplementary Information [file 41467_2025_58211_MOESM1_ESM.pdf]

Supplementary Information

Nb-induced lattice changes to enhance corrosion resistance of $Al_{0.5}Ti_3Zr_{0.5}Nb_xMo_{0.2}$ high-entropy alloys

Xuelian Yu[1], Qingjun Chen[1] *, Xia Cui[1], Delai Ouyang[1]

[1] School of Materials Science and Engineering, Nanchang Hangkong University, Nanchang 330063, China

*Corresponding author: Qingjun Chen, E-mail: qjchen@nchu.edu.cn

Table. S1 Thermodynamic parameters of the high-entropy alloy $Al_{0.5}Ti_3Zr_{0.5}Nb_xMo_{0.2}$.

x	ΔS_{mix} (J/(K· mol))	ΔH_{mix} (kJ/mol)	δ_r (%)	VEC	Ω	$\Delta\chi$ (%)
1	-10.06	-9.13	3.06	4.17	2.27	14.04
0.8	-9.89	-9.8	3.11	4.14	2.05	14.29
0.5	-9.44	-10.97	3.20	4.09	1.71	14.71

Table. S2 Atomic number, electronegativity difference, χ, atomic radius, r, melting point, Tm, density ,ρ, and valence electron concentration ,VEC， of metals.

Element	Al	Ti	Zr	Nb	Mo
Atomic number	13	22	40	41	42
r(Å)	1.432	1.462	1.6	1.46	1.39
T_m (K)	933.5	1941	2125	2741	2883
ρ (g/cm^3)	2.70	4.51	6.51	8.57	10.28
χ	1.61	1.5	1.33	1.6	2.16
VEC	3	4	4	5	6

Table. S3 Electrochemical parameters of polarisation curves of high entropy alloy $Al_{0.5}Ti_3Zr_{0.5}Nb_xMo_{0.2}$ in 3.5% NaCl solution.

**Alloy**	**$E_{corr}(V_{SCE})$**	**$I_{corr}(A/cm^2)$**	**$I_{pass1}(A/cm^2)$**	**$I_{pass2}(A/cm^2)$**	**$E_{pit}(V_{SCE})$**
**$Al_{0.5}Ti_3Zr_{0.5}NbMo_{0.2}$**	-0.0518	3.16×10^{-7}	1.69×10^{-5}	1.46×10^{-5}	8.68
**$Al_{0.5}Ti_3Zr_{0.5}Nb_{0.8}Mo_{0.2}$**	-0.203	3.37×10^{-7}	5.03×10^{-6}	7.07×10^{-6}	8.24
**$Al_{0.5}Ti_3Zr_{0.5}Nb_{0.5}Mo_{0.2}$**	-0.5787	1.11×10^{-6}	2.75×10^{-5}	3.14×10^{-5}	6.9

Table. S4 Valence and relative content of alloying elements in passivation films formed by $Al_{0.5}Ti_3Zr_{0.5}NbMo_{0.2}$ high-entropy alloys at different potentials.

Element	Chemical State	Atomic Concentration(%)							
		$1V_{SCE}$				$4V_{SCE}$			
		d=0nm	2.5nm	10nm	20nm	d=0nm	2.5nm	10nm	20nm
Nb	Nb^{5+}	9.12	7.62	4.86	3.14	8.74	8.76	7.05	6.71
	Nb^{4+}	/	2.06	3.28	/	/		2.32	3.17
	Nb^{2+}	/	/	4.11	8.57	/	/	/	/
Ti	Ti^{4+}	61.82	39.61	27.34	19.11	68.82	53.59	39.43	34.29
	Ti^{3+}	/	21.54	21.85	16.7	/	15.4	22.45	20.37
	Ti^{2+}	/	4.37	13.18	22.24	/	/	4.42	8.21
Zr	Zr^{4+}	9	9.68	11.32	9.43	8.41	8.33	9.46	10.6
	Zr^0	/	/	/	2.55	/	/	/	/
Al	Al^{3+}	11.48	11.09	11	9.61	9.87	11.54	10.75	12.9
	Al^0	1.48	1.08	0.35	3.74	1.42	0.34	1.58	0.47
Mo	Mo^{6+}	5.4	0.41	/	/	2.16	0.88	0.84	0.83
	Mo^{4+}	1.81	2.53	1.02	1.36	0.6	1.16	1.71	2.24
	Mo^0	/	/	3.05	3.45	/	/	/	/

Table. S5 Standard molar generation Gibbs free energy ($\Delta_f G_m^\Theta$) of the relevant oxides at 298.15 K. Changes in Gibbs free energy ($\Delta_r G_m^\Theta$) of generated oxides.

Element	Chemical State	$\Delta_f G_m^\Theta$ kJ/mol	$\Delta_r G_m^\Theta$ kJ/mol
Nb	Nb_2O_5	-1766.0	-580.108
	NbO_2	-740.5	-133.0716
	NbO	-378.6	-141.4216
Ti	TiO_2	-888.8	-414.443
	Ti_2O_3	-1434.2752	-240.913
	TiO	-513.3768	-276.198
Zr	ZrO2	-1042.8	-568.4432
Al	Al_2O_3	-1582.3	-870.7648
Mo	MoO_3	-668.0	43.5352
	MoO_2	-533.0	-29.3216
H	H_2O	-237.1784	/
